# From a hunger-regulating hormone to an antimicrobial peptide: gastrointestinal derived circulating endocrine hormone-peptide YY exerts exocrine antimicrobial effects against selective gut microbiota

**DOI:** 10.1080/19490976.2024.2316927

**Published:** 2024-02-14

**Authors:** Yuchen Xiao, Lingjing Jin, Chao Zhang

**Affiliations:** Fundamental Research Center, Shanghai Yangzhi Rehabilitation Hospital (Shanghai Sunshine Rehabilitation Center), School of Medicine, Tongji University, Shanghai, China

**Keywords:** GI tract, PYY, gut microbiota, antimicrobial peptide, *C. albicans*, Paneth cell

Gut microbes participate in multiple physiological processes, such as host immunity, metabolism, and neurodegeneration. A recent study published in the journal *Science* entitled “Peptide YY: A Paneth cell antimicrobial peptide that maintains Candida gut commensalism” revealed an important finding.^1^ The study identified intestinal-specific Paneth cells (PCs) locally expressing the full-length peptide YY (PYY_1–36_) that acts not only as a precursor of circulating satiety modulating hormone, but also as an antimicrobial peptide (AMP) to maintain the intestinal fungal commensalism by selectively inhibiting *Candida albicans*(*C.* *albicans*) hyphae. This study reveals additional functions of gastrointestinal-derived endocrine hormones beyond their roles in regulating energy homeostasis. It serves as a stimulus for future research to explore the peripheral effects of both endogenous and exogenously administrated circulating peptides on the gut microbiota.

Full-length peptide YY (PYY_1–36_) is a highly conserved 36-amino acid peptide secreted by intestinal endocrine cells (EECs). Once amino acids 1–2 are cleaved by dipeptidyl peptidase IV (DPP-IV), it results in the formation of a circulating hormone (PYY_3–36_).^2^ PYY_3–36_ can cross the blood–brain barrier (BBB) and bind to the neuropeptide Y receptor (Y2) with high affinity in several brain areas that control satiety and act as a satiety hormone that regulate energy balance, food intake, sympathetic vascular tone, digestion, circadian rhythm, and other endocrine and autonomic functions.^3^

In a recent study, Pierre et al. made an interesting finding while investigating enteroendocrine L cells located in the distal mucosa of the mouse ileum. They discovered that Lysozyme-expressing Paneth cells (PCs), recognized for their role in defending the mammalian gut against harmful bacteria, also contain the satiety-regulating peptide PYY, indicating that PYY may have a potential role as an antimicrobial agent. Traditionally, PCs function as immune system defenders in the mammalian gut, which prevent the colonization of inorganic bacteria by secreting several antimicrobial compounds.^4–6^ Therefore, the previous understanding that PYY solely functions as an appetite-modulating hormone seemed incomplete. To test this conjecture, Pierre et al. further investigated the antimicrobial activity of PYY_1–36_ against gram-positive and gram-negative bacteria as well as fungi in vitro. Intriguingly, PYY_1–36_ shares structural similarities with magainin 2, an antimicrobial peptide recognized for its protective function against bacterial and fungal infections in the skin of African clawed frogs.^7,8^ This structural resemblance has attracted further research into the potential inhibitory effect of PYY on the growth and viability of *C. albicans*, the predominant fungus residing in the human gut. *C. albicans* which reside as a commensal yeast in the intestinal tract can transition into a pathogenic hyphal form under specific conditions (e.g., exposing mucins).^9,10^

They also investigated the membrane permeability of *C. albicans* following treatment with PYY_1–36_ and found that Propidium iodide (PI) staining was accumulated in the hyphae. This finding confirmed that PYY_1–36_ exhibited selective antimicrobial activity against *C. albicans* by preferentially inhibiting hyphal growth or inducing hyphal cell death while exerting minimal effect on yeast forms. To further confirm this finding, they conducted cation probe imaging and sulfate charge quenching assays, which further provided evidence to support the cationic specificity of PYY_1–36_ to enable its direct interaction with the surface of *C. albicans* hyphae. Consequently, this interaction not only decreases their activity but also disrupts fungal cell membranes in a manner similar to other antimicrobial peptides (AMPs). In summary, the complete PYY_1–36_ exhibits targeted antimicrobial efficacy against *C. albicans*, particularly in the presence of hyphal forms on mucosal surfaces. This distinguishes its functionality from individuals carrying Lyz1.2 and underscores its distinctive mechanism of action and specificity. Subsequently, *C. albicans* were treated with PYY_1–36_, *in vitro* and *in vivo* to explore the impact of PYY_1–36_ on the intestinal mucosal membrane permeability. The presence of PYY_1–36_ caused a significant reduction in the attachment of *C. albicans* hyphae to the human epithelial cell-line Caco-2, a structurally and functionally similar cell line to tiny intestinal epithelial cells. This demonstrates that PYY_1–36_ can inhibit the adherence of *C.* *albicans* hyphae to intestinal epithelial cells. Furthermore, oral administration of PYY_1–36_ reduced fungal titers in fecal specimen in an animal model of antibiotic-induced chronic *C.albicans* intestinal colonization. On the contrary, the absence of PYY increased fungal colonization, as well as the composition of fungal and bacterial organisms in the mucous layer. Notably, unlike other innate immune AMPs with broad spectrum of action, PC-PYY_1–36_ exhibited microbiological specificity once activated and mucosal compartmentalization within the mucosal environment. The above mechanism allows the host to maintain intestinal fungal homeostasis and hence prevent the development of pathogens.^11^ Overall, these findings highlight the potential role of PYY_1–36_ as a selective antimicrobial peptide against *C. albicans*. It also maintains intestinal fungal balance and prevents uncontrollable fungal overgrowth.

These remarkable findings have reshaped our understanding of the dual functionality of PC-PYY_1–36_, serving as both an innate antimicrobial peptide and a precursor of the anti-satiety circulating hormone. Notably, upon secretion, PYY_1–36_ is swiftly cleaved by the intestinal serine protease DPP-IV into the active form PYY_3–36_, undergoing evaluation in various clinical trials as a potential weight-loss drug.^12^ This study also confirms the therapeutic potential of PYY in the treatment of inflammatory intestinal diseases, such as ileal Crohn’s disease (iCD), where the impaired function of Paneth cells and reduced PYY release coincide with increased pathogenic hyphae in the gut. This, in turn, will trigger immune activation and inflammatory responses (Figure 1). These results demonstrate that PC-PYY is a potential antimicrobial peptide for the treatment of diseases owing to its ability to maintain a symbiotic state in the critical transition zone between the large and small bowel microbiota.

**Figure 1.**
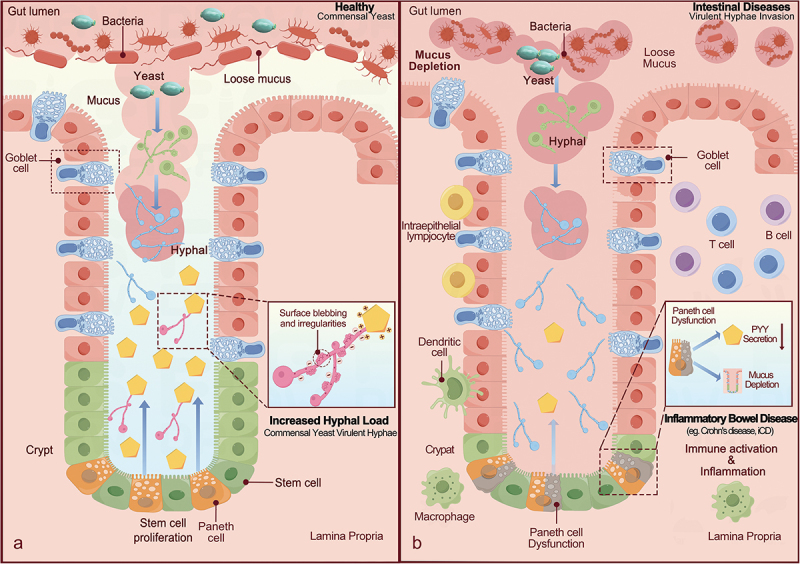
Mechanism of PYY release from paneth cells (PCs), its regulation by fungal symbionts, and their potential implications in inflammatory bowel disease.

(a) In a healthy ileum, symbiotic yeast does not stimulate the release of PYY_1–36_ from PCs. Increased virulent hyphae (blue hyphae) trigger the release of PYY_1–36_ from PCs into the mucus. PYY_1–36_ then targets and eliminates the hyphae (red hyphae), thereby modulating the fungal communities in the gut. (b) In inflammatory bowel disease, such as iCD, elevated mycelial load contributes to immune activation and inflammation. PC dysfunction (represented by brown PCs), is accompanied by a decrease in PYY_1–36_ release or mucus depletion.

In conclusion, PC-PYY is structurally, biologically, and functionally distinct from endocrine peptides and exhibits selective antimicrobial activity against *C. albicans*. It also exerts similar membrane-disrupting effect for different *C. albicans* species. In contrast to broad-spectrum antimicrobial peptides, PYY specifically targets enteropathogenic fungi and contributes to intestinal fungal symbiosis. The regulatory characteristics and specificity of PYY against virulent strains of *C. albicans* differentiate it from other innate immune peptides, indicating that it regulates several aspects of the gut mycobacteria. In ileal Crohn’s disease (iCD), the bioavailability, stability, and bioactivity of PC-PYY are likely to be compromised following Paneth cell dysfunction and mucosal cell depletion, which allows fungi such as *C. albicans* to become virulent, adhesive, and invasive and contribute to the pathogenesis of such disease.

Nonetheless, this study has some limitations. First, a global knockout (KO) of PYY may disrupt its endocrine function and potentially affect the outcome compared to a PC-specific mutant model. To minimize the cumulative metabolic disruption resulting from ubiquitous PYY deficiency, young animals were utilized in the study. Future studies should develop PC-specific PYY mutants to explore its specific physiological functions *in vivo*. Moreover, a high concentration of PYY peptide is utilized in *in vitro* studies, whereas a physiological low concentration is typically present within the gut mucus. Therefore, an *in vitro* study that closely mimics endogenous concentration of PYY may enhance the applicability of these findings in real-life physiological conditions. In conclusion, although this study sheds light on the antimicrobial and regulatory attributes of PYY, these limitations need to be addressed in future research.

Being the main digestion and processing center for food and nutrients, the gastrointestinal (GI) tract secretes multiple peptide hormones, which enters the circulation and regulates the central nervous system to modulate appetite and eating behavior^13,14^ (Figure 2). In addition to PYY, there are other circulating hormones derived from the GI tract which include Gastrin, Ghrelin, Leptin, Somatostatin, Secretin, GIP, CCK, Motilin, GLP-1, GLP-2, OXM, and FGF-19.^15^ The gastrointestinal tract secretes multiple types of peptide hormones into the circulatory system that cross the blood–brain barrier and regulate multiple neuroendocrine circuits in the central nervous system, particularly the hypothalamic region, to modulate appetite and feeding behavior. PYY suppresses appetite by activating POMC/CART neurons and inhibiting NPY/AgRP neurons. Leptin, produced by adipose tissue, contributes to energy balance by activating POMC/CART neurons and inhibiting NPY/AgRP neurons to induce a reduction of food intake. Post-meal released Glucagon-like peptide-1 (GLP-1) decreases food intake by acting on receptors in peripheral tissues and the central nervous system. Cholecystokinin (CCK), released upon dietary fat or protein ingestion, activates CCK-A receptors in brain regions and reduces food intake by inducing feelings of fullness. In turn, ghrelin stimulates feeding behavior before meals by binding to GHSR1α on NPY/AgRP neurons. The complex intricate interplay forms a coordinated neuroregulatory network that induces multiple neuronal activity to modulate appetite and feeding behavior. Several hormones originating from the gastrointestinal tract and other sources exhibit direct or indirect antimicrobial properties. Ghrelin, traditionally recognized for its role in appetite and energy balance regulation, possess antimicrobial properties by inhibiting the growth of *Helicobacter pylori*.^16,17^ The gastrointestinal hormone GLP-2 exerts an inhibitory effect on *Salmonella enterica* and *Helicobacter pylori*.^17^ Insulin, produced by the pancreas to regulate serum glucose homeostasis, enhances immune responses and bacterial clearance via its antimicrobial effects.^18^ Adiponectin, derived from the adipose tissue, regulates antimicrobial peptide production in senescent keratinocytes, thereby protecting against microbial pathogens.^19^ Melatonin, primarily recognized for its involvement in the regulation of the sleep-wake circadian rhythm, contributes to the defense against microbial pathogens due to its antioxidant and immunomodulatory properties.^20–25^ The *in vitro* effect of these hormones on gut bacteria has been explored, but minimal attention has been directed at gut fungi. However, the novel role of PC-PYY as an antimicrobial peptide with selective antifungal activity underscores the importance of Paneth cells and their secretory components in shaping the gut microbiome and providing defense against fungal infections. Investigating the impact of these hormones on gut bacteria has been somewhat limited with few studies on gut fungi. Further, although PYY has been observed in various single-cell RNA-seq datasets, its transcriptional expression is relatively low and exhibits regional variability. A spatial and temporal single cell resolution of PYY mRNA and protein expression requires further evaluation. And the investigation of PYY expressional pattern in different intestine regions could provide more comprehensive insights of its endogenous function.

**Figure 2.**
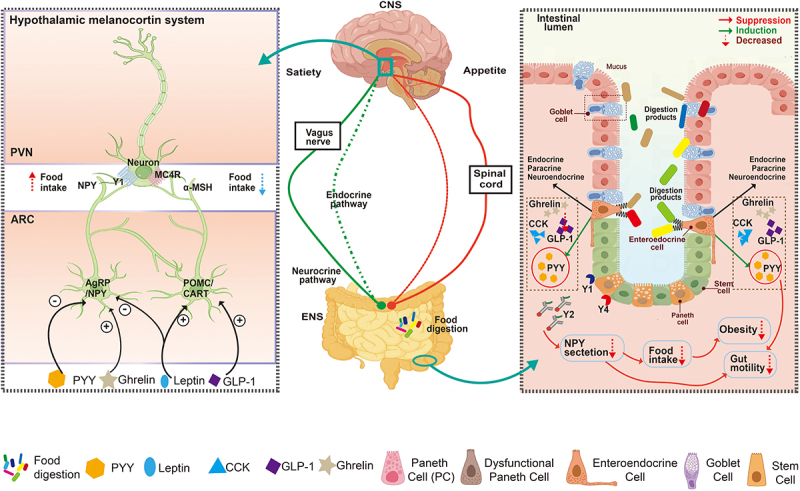
The gut-brain hormone axis: bidirectional hormone signaling between the gut and central nervous system.

The gastrointestinal tract secretes multiple types of peptide hormones into the circulatory system, which pass the blood brain barrier and regulate multiple neuroendocrine circuits in the central nervous system, particularly the hypothalamic region to modulate appetite and eating behavior. AgRP, agouti gene-related protein; ARC, arcuate nucleus; CCK, cholecystokinin; CNS, central nervous system; EECs, enteroendocrine cells; ENS, enteric nervous system; GLP-1, glucagon-like peptide-1; IECs, intestinal epithelial cells; NPY, neuropeptide Y; POMC, proopiomelanocortin; PVN, paraventricular nucleus; PYY, peptide YY.

Overall, this finding expands our understanding of the intricate interplay between the host immune system and the gut microbiome, suggesting that it can potentially bridge between endocrine homeostasis and GI microbes. This also provides a basis for future investigations into the physiological and pathological links between the endocrine and digestive systems, especially the effect of GI-secreted peptide hormones on gut fungus commensalism. Greater emphasis should be placed on addressing various gastrointestinal disorders stemming from microbial dysregulation. Structural modification and optimization of naturally occurring gastrointestinal-secreted peptides, coupled with innovative oral administration approaches, are poised to emerge as effective therapeutic strategies for managing a spectrum of gastrointestinal, immune, and metabolic-related human disorders in the future.

## Data Availability

All of the data generated from this article are all included in this manuscript.
